# Long Non-coding RNA KTN1-AS1 Targets miR-505 to Promote Glioblastoma Progression

**DOI:** 10.1155/2023/4190849

**Published:** 2023-01-31

**Authors:** Kai Guo, Lingling Fang, Mingjian Li, Aizheng Li, Na Liu

**Affiliations:** ^1^Department of Neurosurgery, Weifang People's Hospital, Weifang, Shandong 261000, China; ^2^Department of Neurology, Anqiu People's Hospital, Anqiu, Shandong 262100, China

## Abstract

Glioblastoma (GBM) is a highly malignant cancer, the prognosis of which is pretty poor. Long non-coding RNAs (lncRNAs) are a class of non-coding RNAs, which play important roles in carcinogenesis process of many cancers including GBM. In this study, we want to clarify the expression, biological function, and molecular mechanism of lncRNA KTN1 antisense RNA 1 (KTN1-AS1) in GBM tumor progression. We found that KTN1-AS1 expression was upregulated in GBM tissues and cell lines. KTN1-AS1 played oncogenic roles to facilitate proliferation, migration, and invasion of GBM cells. Then, we revealed that miR-505 was a target of KTN1-AS1, and its expression was decreased in GBM. KTN1-AS1 contributed to GBM progression by mediating miR-505. Finally, we demonstrated that KTN1-AS1 upregulated some target oncogenes of miR-505 including ZEB2, HMGB1, and RUNX2 in GBM cells. All in all, we concluded that the highly expressed KTN1-AS1 in GBM played oncogenic roles to facilitate GBM progression by targeting miR-505.

## 1. Introduction

Glioma is the most common primary tumor of the central nervous system, and glioblastoma (GBM) is the most malignant subtype of glioma, which is characterized by an unacceptably high invasive potential [1, 2]. Despite advanced therapies such as surgery, chemotherapy, and radiotherapy have been widely applied in the clinical treatment at present, the prognosis of GBM patients remains poor due to the high malignancy [3, 4]. Therefore, it is vital to study the mechanisms underlying the invasive phenotype of GBM and identify novel therapeutic targets to improve prognosis.

Long non-coding RNAs (lncRNAs) are a class of non-coding RNAs with more than 200 nucleotides [5]. More and more studies indicate that lncRNAs affect a variety of biological processes and play important roles in carcinogenesis process of many cancers including GBM [6, 7]. LncRNAs are able to play biological roles via multiple mechanisms, and the most common one is the competitive endogenous RNAs (ceRNAs) way. In this way, lncRNAs increase the expression of microRNAs (miRNAs) target genes by competitively sponging these miRNAs [8, 9].

LncRNA KTN1 antisense RNA 1 (KTN1-AS1) is a newly identified lncRNA, which has already been reported as an oncogenic gene in several malignant tumors [10–12]. However, to our knowledge, the expression, biological function, and molecular mechanism of KTN1-AS1 in GBM remain still unknown. Therefore, in this study, we want to focus on KTN1-AS1 to clarify its roles in GBM tumor progression.

## 2. Materials and Methods

### 2.1. Tissue Samples

Paired GBM tissues and normal paratumor tissues were collected from 40 patients diagnosed with GBM in Weifang People's Hospital. None of these patients received local or systemic treatment before surgery. This study was approved by the Ethics Committee of Weifang People's Hospital. All participants enrolled in this study had signed written informed consent.

### 2.2. Cell Culture and Transfection

Human GBM cell lines (A172, U87, U251, and LN229) and normal human astrocytes (NHA) were obtained from American Type Culture Collection (ATCC, USA). All these cell lines were cultured in dulbecco's modified eagle medium (DMEM) medium (Gibco, USA) containing 10% fetal bovine serum (FBS) at 37°C with 5% CO_2_. Plasmids, siRNAs, miR-505 mimics, miR-505 inhibitors, or corresponding negative controls (NC) were transfected into cells using Lipofectamine 3000 (Invitrogen, USA).

### 2.3. RT-qPCR

Total RNAs were extracted using TRIzol Reagent (Invitrogen) and reversely transcribed into cDNAs using QuantiTect Reverse Transcription Kit (Qiagen, Germany). Real-time reverse transcription polymerase chain reaction (qRT-PCR) reaction was conducted using SYBR Green qPCR mix kit (Takara, Japan). Glyceraldehyde-3-phosphate dehydrogenase (GAPDH) and U6 were selected as internal references for quantifying relative expression of lncRNAs and miRNAs, respectively. The relative gene expression was calculated with the 2^−*ΔΔ*Ct^ method. Primer sequences for KTN1-AS1 were, forward: 5′-AGGGAAATTTGGGCAGAAGT-3′ and reverse: 5′-GTTACCCGTGTGAGCCTGAT-3′.

### 2.4. Western Blot Assay

Total proteins were extracted using Radio Immunoprecipitation Assay (RIPA) buffer (Beyotime, China) containing protease inhibitor and qualified by a BCA Protein Assay Kit (Thermo Scientific, USA). Proteins were separated by 10% sodium dodecyl sulfate-polyacrylamide gel electrophoresis (SDS-PAGE) gel and transferred onto polyvinylidene fluoride (PVDF) membranes (Millipore, USA). The membranes were then reacted with specific primary antibodies against ZEB2, HMGB1, RUNX2 (ab138222, ab18256, ab76956, Abcam, USA), and GAPDH (ab8245, Abcam) at 4°C overnight and a secondary antibody at room temperature for 2 hours. Band exposure was performed in the enhanced chemiluminescence (ECL) detection system (Pierce, USA).

### 2.5. CCK-8 Assay

Cells were seeded in a 96-well plate (1 × 10^3^ cells/well) 24 hours before transfection. At 24, 48, or 72 hours after transfection, 10 *μ*L Cell Counting Kit-8 (CCK-8) solution was added into each well, and absorbance at 450 nm of each well was recorded after incubating for 1 hour at 37°C.

### 2.6. 5-Ethynyl-2′-Deoxyuridine (EdU) Assay

Cells were seeded in a 24-well plate (1 × 10^5^ cells/well) 24 hours before transfection. At 48 hours after transfection, Cell-light EdU Apollo 567 In Vitro Imaging Kit (Ribobio, China) was used to perform EdU assay according to the manufacturer's instructions.

### 2.7. Wound-Healing Assay

Cells were seeded in a 24-well plate (1 × 10^5^ cells/well) and cultured until 90% integration. Confluent monolayers were scratched by using a pipette tip. Images were captured at 0 and 24 hours after culturing with serum-free medium.

### 2.8. Transwell Assay

Transwell assay was conducted using Matrigel coated chamber (Corning, USA). Cells were transfected for 48 hours and harvested to seed into the upper chamber with serum-free medium. Medium containing 10% FBS was added into the lower chamber. After 24 hours, cells invaded through the membrane were fixed with methanol and stained with 0.1% crystal violet to count cell numbers.

### 2.9. Luciferase Reporter Assay

KTN1-AS1 sequences containing wild-type (wt) and mutant-type (mut) miR-505 binding sites were inserted into a pGL4 vector to construct KTN1-AS1-wt and KTN1-AS1-mut plasmids. Cells were co-transfected with KTN1-AS1-wt or KTN1-AS1-mut plasmids and miR-505 mimic. Luciferase activity was analyzed using the dual-luciferase reporter assay system (Promega, USA).

### 2.10. Statistical Analysis

All experiments were carried out at least three times, and data were presented as mean ± SD. The differences between groups were assessed by one-way Analysis of Variance (ANOVA) or Student's *t* test using GraphPad Prism Software (V5.0, GraphPad Software, USA). *P* values less than 0.05 were considered statistically significant.

## 3. Results

### 3.1. KTN1-AS1 Expression Is Upregulated in GBM Tissues and Cell Lines

**Figure 1:**
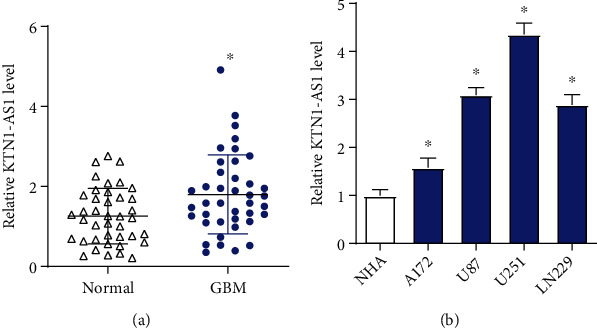
KTN1-AS1 expression is upregulated in GBM tissues and cell lines. (a) KTN1-AS1 expression was detected in GBM tissues and adjacent normal tissues by qRT-PCR. (b) KTN1-AS1 expression was detected in GBM cell lines and NHA cells by qRT-PCR. ∗*P* < 0.05.

In order to explore the expression profile of KTN1-AS1 in GBM, qRT-PCR was applied to measure KTN1-AS1 expression in GBM tissues and cell lines. The result showed that KTN1-AS1 expression was higher in GBM tissues compared with adjacent normal tissues (Figure 1(a)). Moreover, the expression of KTN1-AS1 in GBM cell lines (A172, U87, U251, and LN229) was also higher than that of in NHA cells (Figure 1(b)). All these results indicated an increased KTN1-AS1 expression in GBM tissues and cell lines.

### 3.2. KTN1-AS1 Facilitates Proliferation, Migration, and Invasion in GBM Cells

**Figure 2:**
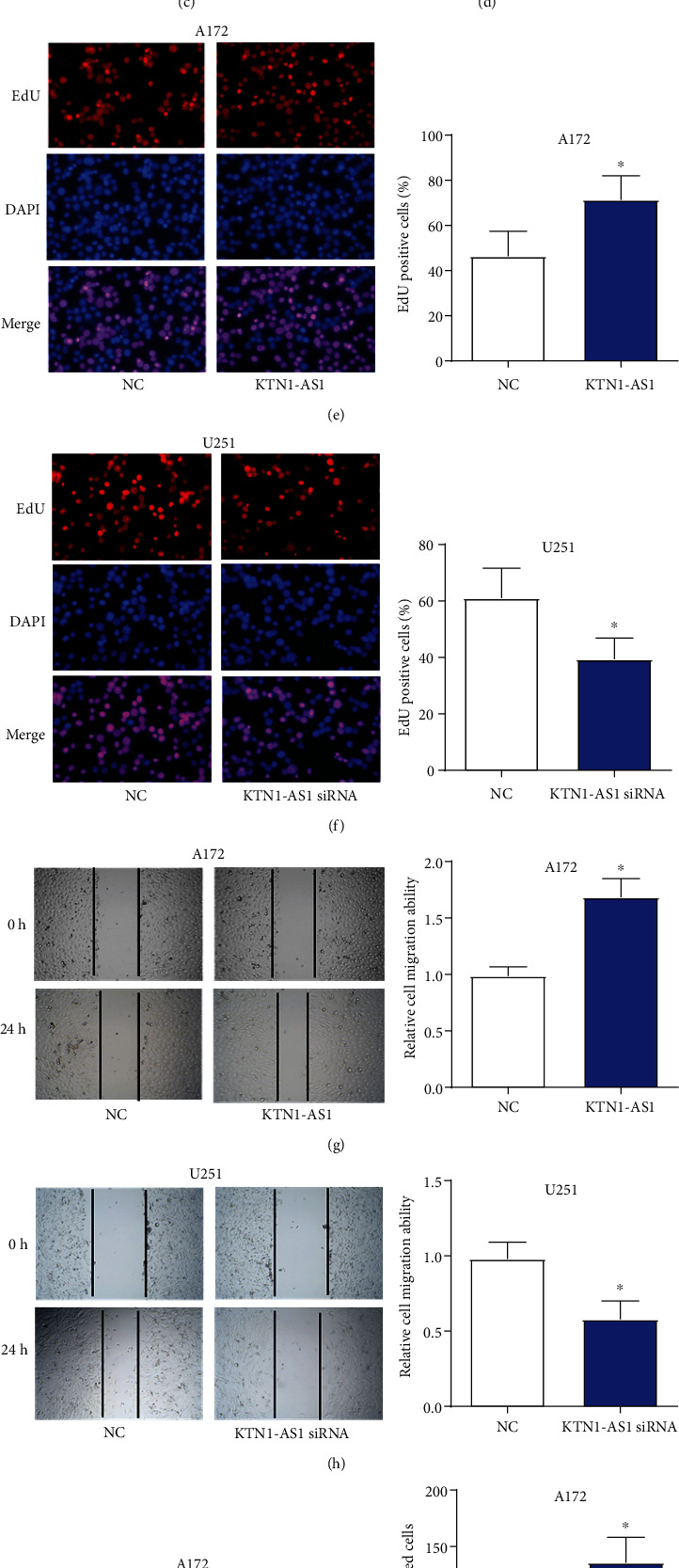
KTN1-AS1 facilitates proliferation, migration, and invasion in GBM cells. (a) and (b) Overexpressing or silencing efficiency of KTN1-AS1 in GBM cells was assessed by qRT-PCR. (c) and (d) Proliferation of GBM cells were assessed by CCK8 assays. (e) and (f) Proliferation of GBM cells were assessed by EdU assays. (g) and (h) Migration of GBM cells were assessed by wound-healing assays. (i) and (j) Invasion of GBM cells were assessed by transwell assays. ∗*P* < 0.05.

To investigate the biological functions of KTN1-AS1 in GBM cells, first, KTN1-AS1 was upregulated in A172 cells by transfecting KTN1-AS1 overexpression plasmids and silenced in U251 cells by transfecting KTN1-AS1 siRNAs (Figures 2(a) and 2(b)). Then, CCK-8 assay and EdU assay found that overexpressing KTN1-AS1 significantly enhanced the proliferative ability of A172 cells, and silencing KTN1-AS1 obviously inhibited the proliferation of U251 cells (Figures 2(c), 2(d), 2(e), and 2(f)). Accordingly, wound-healing assay and transwell assay displayed that overexpressing KTN1-AS1 significantly promoted the migration and invasion of A172 cells, and silencing KTN1-AS1 suppressed the migration and invasion of U251 cells (Figures 2(g), 2(h), 2(i), and 2(j)). These results implied that KTN1-AS1 played oncogenic roles to facilitate malignant processes of GBM cells.

### 3.3. MiR-505 Is a Target of KTN1-AS1

**Figure 3:**
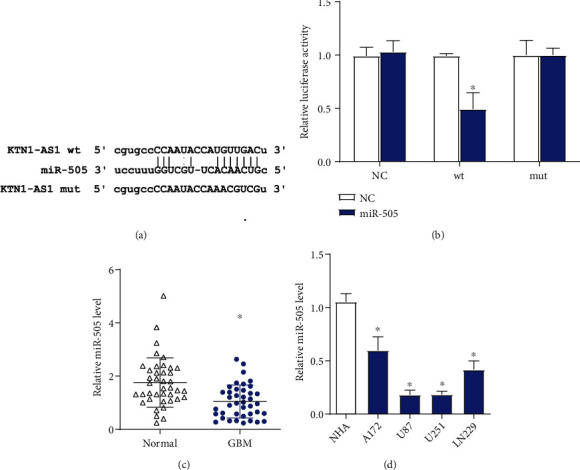
miR-505 is a target of KTN1-AS1. (a) Predicted binding site of miR-505 in KTN1-AS1 sequence. (b) The predicted binding site was subsequently verified by luciferase reporter assay. (c) miR-505 expression was detected in GBM tissues and adjacent normal tissues by qRT-PCR. (d) miR-505 expression was detected in GBM cell lines and NHA cells by qRT-PCR. ∗*P* < 0.05.

To investigate the regulatory mechanism of KTN1-AS1, we used StarBase database to find the target miRNAs of KTN1-AS1. MiR-505, which had been reported to function as a tumor suppressor gene, was chosen for validation. The predicted binding site (Figure 3(a)) was subsequently verified by luciferase reporter assay (Figure 3(b)). We also demonstrated that miR-505 was significantly downregulated in GBM tissues and cell lines (Figures 3(c) and 3(d)). These data showed that miR-505 was a target of KTN1-AS1, and its expression was decreased in GBM.

### 3.4. KTN1-AS1 Contributes to GBM Progression by Mediating miR-505

**Figure 4:**
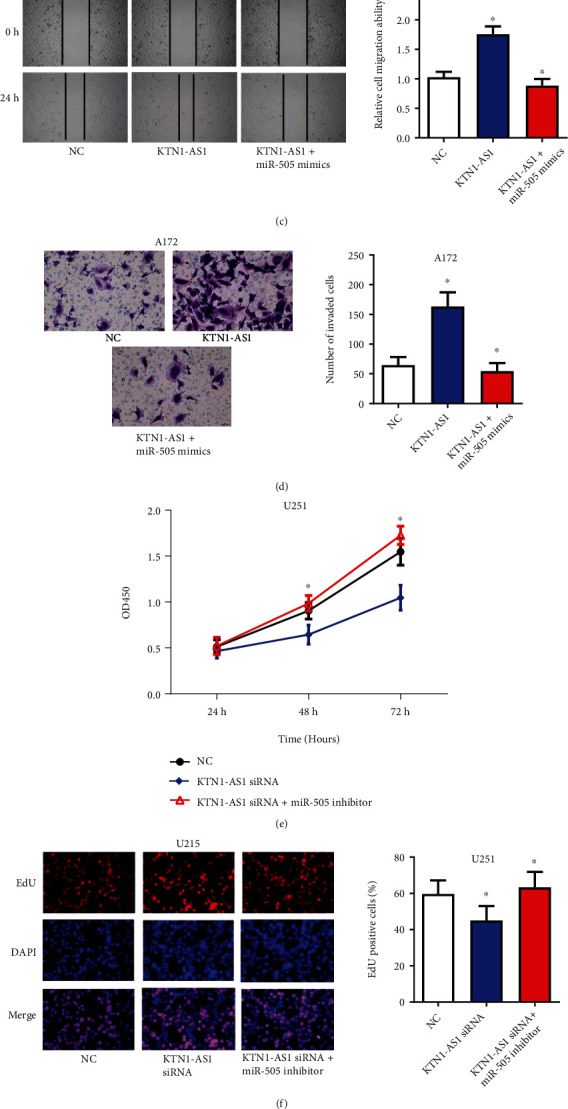
KTN1-AS1 contributes to GBM progression by mediating miR-505. (a)–(d) Effect of overexpressing KTN1-AS1 and miR-505 on A172 cells proliferation, migration, and invasion was evaluated by CCK8 assays (a), EdU assays (b), wound-healing assays (c), and transwell assays (d). (e)–(h) Effect of silencing KTN1-AS1 and miR-505 on U251 cells proliferation, migration, and invasion was evaluated by CCK8 assays (e), EdU assays (f), wound-healing assays (g), and transwell assays (h). ∗*P* < 0.05.

Then, we further determined the roles of miR-505 in KTN1-AS1 induced GBM progression. Our data showed that overexpression of miR-505 significantly reversed the KTN1-AS1 caused proliferation, migration, and invasion acceleration in A172 cells (Figures 4(a), 4(b), 4(c), and 4(d)). Similarly, inhibition of miR-505 rescued the proliferation, migration, and invasion suppression induced by KTN1-AS1 silence in U251 cells (Figures 4(e), 4(f), 4(g), and 4(h)). The above data suggested that KTN1-AS1 contributed to GBM progression by mediating miR-505.

### 3.5. KTN1-AS1 Upregulates Target Oncogenes of miR-505

**Figure 5:**
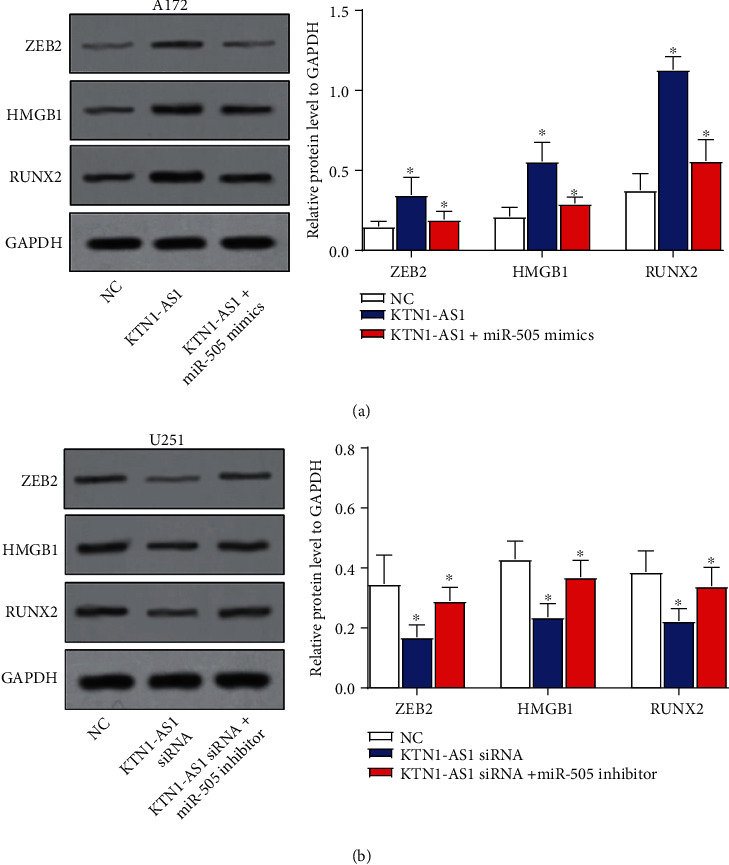
KTN1-AS1 upregulates target oncogenes of miR-505. (a) Effect of overexpressing KTN1-AS1 and miR-505 on the expression of ZEB2, HMGB1, and RUNX2 in A172 cells was detected by western blot assays. (b) Effect of silencing KTN1-AS1 and miR-505 on the expression of ZEB2, HMGB1, and RUNX2 in A172 cells was detected by western blot assays. ∗*P* < 0.05.

Finally, we studied the regulation of KTN1-AS1 on some target genes of miR-505 including ZEB2, HMGB1, and RUNX2 in GBM cells. Our data showed that the expression of these oncogenes was significantly raised by KTN1-AS1, which could be attenuated by miR-505 in A172 cells (Figure 5(a)). Accordingly, the expression of these oncogenes significantly decreased after KTN1-AS1 silencing, which could be reversed by miR-505 inhibition in U251 cells (Figure 5(b)). The above data suggested that KTN1-AS1 upregulated these target oncogenes of miR-505 by mediating miR-505.

## 4. Discussion

GBM is generally considered as a very infiltrative and malignant brain tumor, and its pathogenesis involves the dysregulation of many oncogenes and tumor suppressor genes [13]. More and more evidences have proved that lncRNAs participate in the pathogenesis of many cancers, including GBM [14, 15]. Although many research has been conducted to uncover the potential roles of lncRNAs in GBM progression, while only limited lncRNAs have been reported, and the functions of most lncRNAs remain unclear in GBM. KTN1-AS1 is a novel lncRNA, which has been reported to be upregulated and exert carcinogenesis in some other cancers but not in GBM [10–12]. Hence, in our present research, the expression, biological function, and molecular mechanism of KTN1-AS1 in GBM were investigated.

First, we showed that KTN1-AS1 expression was higher in GBM tissues than that of in adjacent normal tissues and was higher in GBM cell lines than that of in NHA cells. We concluded that KTN1-AS1 expression was increased in GBM tissues and cell lines, indicating that it might play oncogenic roles in GBM. Subsequently, we conducted in vitro experiments and found that overexpressing KTN1-AS1 in GBM cells was able to promote proliferation, migration, and invasion, while silencing KTN1-AS1 have the reverse impacts. So we considered that the highly expressed KTN1-AS1 in GBM played oncogenic roles to facilitate malignant processes of GBM cells.

LncRNAs always exert their roles by binding with miRNAs [8, 9]. Herein, we predicted that miR-505 might bind to KTN1-AS1 and then verified it by luciferase reporter assay. We also demonstrated that miR-505 was significantly downregulated in GBM tissues and cell lines. According to many studies, miR-505 has been proved to act as a tumor suppressor gene in some cancers, including pancreatic cancer [16], prostate cancer [17], non-small cell lung cancer [18], hepatocellular carcinoma [19], and so on. In GBM, miR-505 could inactivate the Wnt/*β*-catenin signaling pathway by directly targeting and inhibiting WNT7B to inhibit tumorigenesis as a tumor suppressor [20]. Then, we further determined that overexpression of miR-505 significantly reversed the effects of KTN1-AS1, and inhibition of miR-505 rescued the influence of KTN1-AS1 silence in GBM cells. So we concluded that KTN1-AS1 contributed to GBM progression by mediating miR-505.

Because lncRNAs could increase the expression of miRNAs target genes in the ceRNA manner, so we explored the downstream genes of KTN1-AS1/miR-505 axis. We displayed that the expression of some validated miR-505 targeted oncogenes genes including ZEB2, HMGB1, and RUNX2 [21–23] was significantly raised by KTN1-AS1 and downregulated by miR-505. ZEB2 is a transcription factor that mainly promotes epithelial-to-mesenchymal transition and regulating differentiation, cancer stem cell-like traits, apoptosis, cell cycle arrest, and metastasis of cancers [24]. Aberrant HMGB1 expression has been reported in different types of cancers, and many studies also verified the promoting role of HMGB1 in the genesis of some cancers including GBM [25, 26]. RUNX2 level has been reported to be upregulated in GBM, and the cross-talk between Cyclic 3′,5′-adenosinemonophosphate/protein kinase A (c-AMP/PKA) signaling and RUNX2 can promote the malignant phenotype of GBM cells [27]. We think that KTN1-AS1 may play oncogenic roles by upregulating these oncogenes by mediating miR-505. Therefore, targeting KTN1-AS1 or overexpression of miR-505 may have potential anti-tumor effect, which are important theoretical basis for clinical implications.

In conclusion, this study presented that lncRNA KTN1-AS1 was upregulated in GBM and played oncogenic roles to facilitate proliferation, migration, and invasion of GBM cells by targeting miR-505. KTN1-AS1 could serve as an underlying therapeutic target for GBM.

## Data Availability

Data supporting this research article are available from the corresponding author or first author on reasonable request.
